# Decreased Expression Of apM1 in Omental and Subcutaneous Adipose Tissue of Humans With Type 2 Diabetes

**DOI:** 10.1155/EDR.2000.81

**Published:** 2000

**Authors:** Michael A. Statnick, Lisa S. Beavers, Laura J. Conner, Helena Corominola, Dwayne Johnson, Craig D. Hammond, Ronit Rafaeloff-Phail, Thomas Seng, Todd M. Suter, James P. Sluka, Eric Ravussin, Robert A. Gadski, Jose F. Caro

**Affiliations:** 1 Lilly Research Laboratories Lilly Corporate Center Indianapolis IN 46285-0424 USA

## Abstract

We have screened a subtracted cDNA library in order to identify differentially expressed genes in omental adipose tissue of human patients with Type 2 diabetes. One clone (#1738) showed a marked reduction in omental adipose tissue from patients with Type 2 diabetes. Sequencing and BLAST analysis revealed clone #1738 was the adipocyte-specific secreted protein gene apM1 (synonyms ACRP30, AdipoQ, GBP28). Consistent with the murine orthologue, apM1 mRNA was expressed in cultured human adipocytes and not in preadipocytes. Using RT-PCR we confirmed that apM1 mRNA levels were significantly reduced in omental adipose tissue of obese patients with Type 2 diabetes compared with lean and obese normoglycemic subjects. Although less pronounced, apM1 mRNA levels were reduced in subcutaneous adipose tissue of Type 2 diabetic patients. Whereas the biological function of apM1 is presently unknown, the tissue specific expression, structural similarities to TNFα and the dysregulated expression observed in obese Type 2 diabetic patients suggest that this factor may play a role in the pathogenesis of insulin resistance and Type 2 diabetes.


*Int. Jnl. Experimental Diab. Res.*, Vol. 1, pp. 81–88
Reprints available directly from the publisher
Photocopying permitted by license only

© 2000 OPA (Overseas Publishers Association) N.V.
Published by license under
the Harwood Academic Publishers imprint,
part of The Gordon and Breach Publishing Group.
Printed in Malaysia.

### Decreased Expression of apM1 in Omental and Subcutaneous Adipose Tissue of Humans with Type 2 Diabetes

MICHAEL A. STATNICK*, LISA S. BEAVERS, LAURA J. CONNER, HELENA COROMINOLA, DWAYNE JOHNSON, CRAIG D. HAMMOND, RONIT RAFAELOFF-PHAIL, THOMAS SENG, TODD M. SUTER, JAMES P. SLUKA, ERIC RAVUSSIN, ROBERT A. GADSKI and JOSE F. CARO

*Lilly Research Laboratories, Lilly Corporate Center, Indianapolis, IN 46285-0424, USA*

*(Received 28 September 1999; In final form 18 January 2000)*

**We have screened a subtracted cDNA library in order to identify differentially expressed genes in omental adipose tissue of human patients with Type 2 diabetes. One clone (#1738) showed a marked reduction in omental adipose tissue from patients with Type 2 diabetes. Sequencing and BLAST analysis revealed clone #1738 was the adipocyte-specific secreted protein gene apM1 (synonyms ACRP30, AdipoQ, GBP28). Consistent with the murine orthologue, apM1 mRNA was expressed in cultured human adipocytes and not in preadipocytes. Using RT-PCR we confirmed that apM1 mRNA levels were significantly reduced in omental adipose tissue of obese patients with Type 2 diabetes compared with lean and obese normoglycemic subjects. Although less pronounced, apM1 mRNA levels were reduced in subcutaneous adipose tissue of Type 2 diabetic patients. Whereas the biological function of apM1 is presently unknown, the tissue specific expression, structural similarities to TNFα, and the dysregulated expression observed in obese Type 2 diabetic patients suggest that this factor may play a role in the pathogenesis of insulin resistance and Type 2 diabetes.**

*Keywords:* Adiponectin, Acrp30, GBP28, AdipoQ, subtractive hybridization, differential gene expression

### INTRODUCTION

Obesity is a major health problem in industrialized countries, and is accompanied by a host of clinical conditions including insulin resistance, Type 2 diabetes, hypertension, and vascular disease (collectively referred to as Syndrome X). However, the molecular and pathophysiological mechanisms of obesity, insulin resistance, and Type 2 diabetes are poorly understood. Several tissues appear to be central to the pathogenesis of obesity and Type 2 diabetes including the brain, adipose tissue, skeletal muscle, and liver.

Adipose tissue is highly specialized, and plays important roles in glucose homeostasis, lipid synthesis and metabolism, and energy storage.[1,2] More recently, the role of adipose tissue as a passive storage depot has changed to that of a true endocrine organ. Several proteins important in the regulation of glucose and fatty acid metabolism are secreted by adipocytes.

*Corresponding author. Endocrine Research, Lilly Research Laboratories, Lilly Corporate Center, Indianapolis, IN 46285-0424. Tel.: 317-277-1123, Fax: 317-276-9086, e-mail: m.statnick@lilly.com

For example, tumor necrosis factor $\alpha$ (TNF$\alpha$) is secreted by adipocytes and is elevated in adipose tissue and circulating plasma of humans with obesity.[3] Interestingly, TNF$\alpha$ interferes with insulin receptor signaling and inhibits insulin-stimulated glucose transport[4] providing a possible mechanism of obesity related insulin resistance. Leptin is another cytokine secreted from adipocytes that in animal models has been shown to regulate appetite, energy expenditure, and adiposity.[5] Lastly, plasminogen activator inhibitor-1 (PAI-1) is secreted from adipose tissue. Both mRNA and plasma levels of PAI-1 were found to be positively correlated with the amount of omental/intra-abdominal adiposity.[6,7] Thus, through the secretion of these, and possibly other, cytokines adipose tissue actively participates in the pathogenesis of Syndrome X.

Recently, another novel adipose-specific gene was isolated based on differential expression from 3T3-L1 adipoctyes,[8,9] and was later found in human adipose tissue.[10] ApM1 (synonyms: Acrp30, AdipoQ, GBP28), encodes a 28–30 kDa secreted protein that is structurally similar to complement factor C1q[8], and TNF$\alpha$.[11] In the present study, we analyzed a subtracted cDNA library from omental adipose tissue of human patients with Type 2 diabetes to identify differentially expressed genes in human adipose associated with diabetes. Herein we report that apM1 mRNA is reduced in omental and to a lesser degree in subcutaneous adipose tissue of human Type 2 diabetic patients. These findings provide additional data characterizing apM1 expression and suggest a potential role for apM1 in insulin resistance and Type 2 diabetes.

### MATERIALS AND METHODS

### Materials

Abdominal omental and subcutaneous adipose tissue was collected at Eastern Carolina University Medical Center from patients undergoing gastric bypass surgery (obese non-diabetic and obese Type 2 diabetic subjects) or elective abdominal surgery (lean non-diabetic subjects). Informed consent was obtained from all patients used in the study prior to tissue collection, and the protocols approved by the Institutional Review Board at Eastern Carolina University. Donors were categorized as lean non-diabetic, obese non-diabetic or obese Type 2 diabetic based on presurgical examinations and records (Tab. I). Human preadipocytes and differentiated adipocytes (ZenBio, Research Triangle Park, NC) were isolated from subcutaneous adipose tissue from 1 male and 2 female patients (age = 33–55, BMI = 20.9–25.1). The cells were cultured under 95%/5% $O_2/CO_2$, at 37°C in DMEM/Ham's F10 (1:1) containing 3% fetal calf serum, 60 U/ml penicillin/streptomycin, 25 µg/ml fungizone with or without 100 nM insulin and 1 µM dexamethasone (for adipocytes and preadipocytes, respectively).

### Generation and Screening of Subtracted cDNA Libraries

Total RNA was isolated from omental adipose tissue from 4 obese non-diabetic and 4 obese Type 2 diabetic female patients (Tab. I) using 4 M guanidine isothiocyanate solution (Gibco, BRL) and phenol : choloroform extraction. cDNA was synthesized from total RNA using SMART™ cDNA synthesis kit (Clontech, Palo Alto, CA), and a subtracted cDNA library was generated for obese non-diabetic minus obese diabetic omental adipose tissue by Suppression PCR™ (Clontech). Approximately 9200 independent clones were randomly picked into 96-well plates (alternately, plasmid cDNA of the clones was alkaline denatured) and arrayed in duplicate onto charged nylon membranes (Amersham, Piscataway, NJ) using a PBA Flexys robot (Genomic Solutions, Ann Arbor, MI). Clones were identified by differential hybridization to $^{32}$P-labeled cDNA probes generated by reverse

TABLE I Clinical characteristics of human adipose tissue donors used in the analysis of apM1 expression

| | $n$ | Age (years) | BMI (kg/m$^2$) | Fasting Plasma Glucose (mg/dl) | Fasting Plasma Insulin (μU/ml) |
|---|---|---|---|---|---|
| Subtracted Library | | | | | |
| Obese non-diabetic | 4 | 50.5 ± 3.3 | 52.9 ± 4.2 | 103 ± 1.9 | 17.5 ± 5.8 |
| Type 2 diabetic | 4 | 48.5 ± 4.2 | 51.3 ± 6.1 | 175 ± 22.5* | 32.3 ± 7.2 |
| Omental Adipose Tissue | | | | | |
| Lean | 6 | 37.7 ± 4.1 | 22.2 ± 1.3[a] | ND | ND |
| Obese non-diabetic | 10 | 37.5 ± 4.8 | 51.5 ± 4.0[b] | 97.3 ± 3.2 | 21.7 ± 4.0 |
| Type 2 diabetic | 8 | 45.1 ± 2.7 | 53.7 ± 3.1[b] | 185.7 ± 19.4** | 29.2 ± 6.1 |
| Subcutaneous Adipose Tissue | | | | | |
| Lean | 7 | 55.1 ± 7.5 | 22.5 ± 0.9[a] | ND | ND |
| Obese non-diabetic | 9 | 40.2 ± 5.6 | 51.1 ± 3.9[b] | 89.9 ± 5.1 | 17.4 ± 3.8 |
| Type 2 diabetic | 8 | 46.7 ± 2.2 | 47.3 ± 3.5[b] | 183.6 ± 21.3** | 33.2 ± 8.7 |

[a] Data are shown as means ± SEM.
[b] $P \leq 0.001$ one-way ANOVA with Tukey post hoc test.
* $P \leq 0.01$ Student's *t*-test.
** $P \leq 0.0001$ Student's *t*-test.
ND = not determined.

transcription of 10 ug total RNA (isolated from the same patients used to construct the library) using an Oligo *dT* primer and Superscript II™ reverse transcriptase (Gibco BRL). Putative differentially expressed clones were sequenced, and subjected to BLAST analysis.

### Semi-quantitative Reverse Transcription Polymerase Chain Reaction (RT-PCR)

First strand cDNA was prepared in duplicate from 0.1, 0.5 and 1.0 ug of DNaseI treated total RNA using Oligo *dT* and Superscript II™ reverse transcriptase (Gibco BRL). Primer concentration, annealing temperature, template concentration, and cycle number were optimized for apM1 and rS9 amplifications. Duplicate PCR reactions (50 μl) were incubated at 94°C 2 min (to denature TaqStart antibody), and then cycled at 94°C 30 s, 60°C 30 s, 72°C 1 min. Aliquots were taken from each reaction during exponential amplification at 23, 27 and 30 cycles. Quantification of PCR products was based on Southern blots of the reactions using internal 32P-labeled oligonucleotide probes to apM1 and rS9. Replication of experimental results in omental and subcutaneous adipose tissue was performed for each patient using identical conditions on independently prepared samples of cDNA as above. To test whether genes identified by differential hybridization were expressed in adipocytes (as opposed to macrophages or vascular endothelial cells associated with adipose tissue), 1 ug of DNaseI treated total RNA pooled from 3 patients was reverse transcribed to cDNA. ApM1 and rS9 were amplified by PCR for 30 cycles using identical conditions as above.

### Image and Statistical Analysis

Image analysis for all hybridization studies was performed using a PersonalFX phosphorimager and QuantityOne software (BioRad, Hercules, CA) or AIS (Imaging Research Inc., Ontario, Canada) imaging software for colony arrays. Normalized apM1 levels were compared by one-way ANOVA with post hoc tests (for multiple group comparisons) or Student's *t*-test (for comparisons between two groups).

### RESULTS

In order to identify novel genes that are differentially expressed in adipose tissue of human

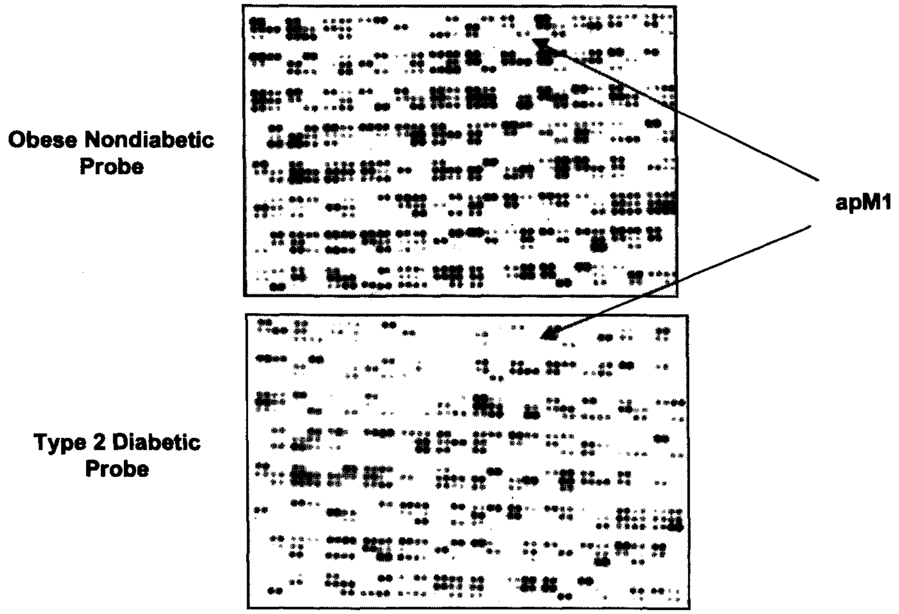

FIGURE 1 High-density analysis of differential gene expression in omental adipose tissue from obese non-diabetic and obese Type 2 diabetic patients. Analysis of differential gene expression on cDNA arrays identified apM1 as a potential regulated gene. Both blots were hybridized to Oligo *dT*-primed $^{32}P$-labeled first strand cDNA synthesized from 10 μg total RNA isolated from obese non-diabetic or obese Type 2 diabetic patients, respectively, normalized to cpm probe/ml hybridization buffer.

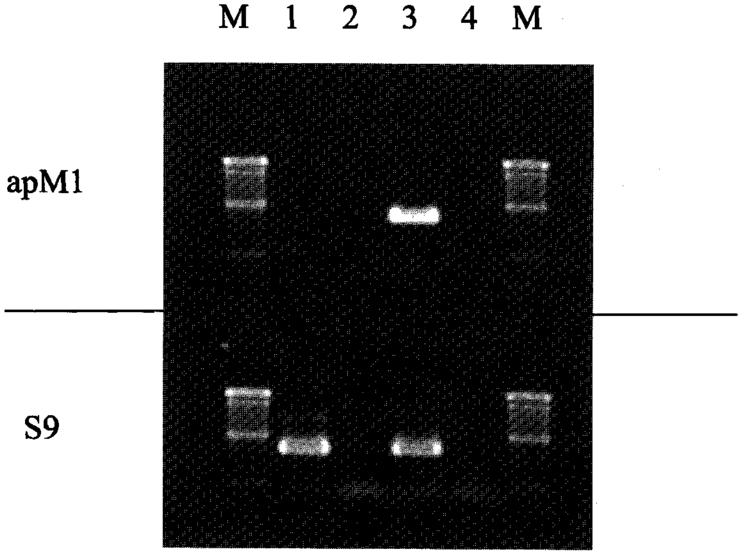

FIGURE 2 Expression of apM1 mRNA by RT-PCR in human preadipocytes and mature adipocytes. Amplification of apM1 or ribosomal S9 (control) was performed using Oligo *dT*-primed cDNA synthesized from 1 μg DNaseI treated total RNA following 30 cycles of PCR. Reverse transcriptase negative samples were used to assess contamination by genomic DNA. (M) 100bp DNA ladder, (1) preadipocyte cDNA, (2) preadipocyte RT-control, (3) adipocyte cDNA, (4) adipocyte RT-control.

patients with Type 2 diabetes, we randomly screened a subtracted cDNA library enriched in mRNAs overexpressed in obese non-diabetic patients. Using differential hybridization of colony/cDNA arrays, one clone (#1738) consistently showed a marked reduction (approximately 4–6

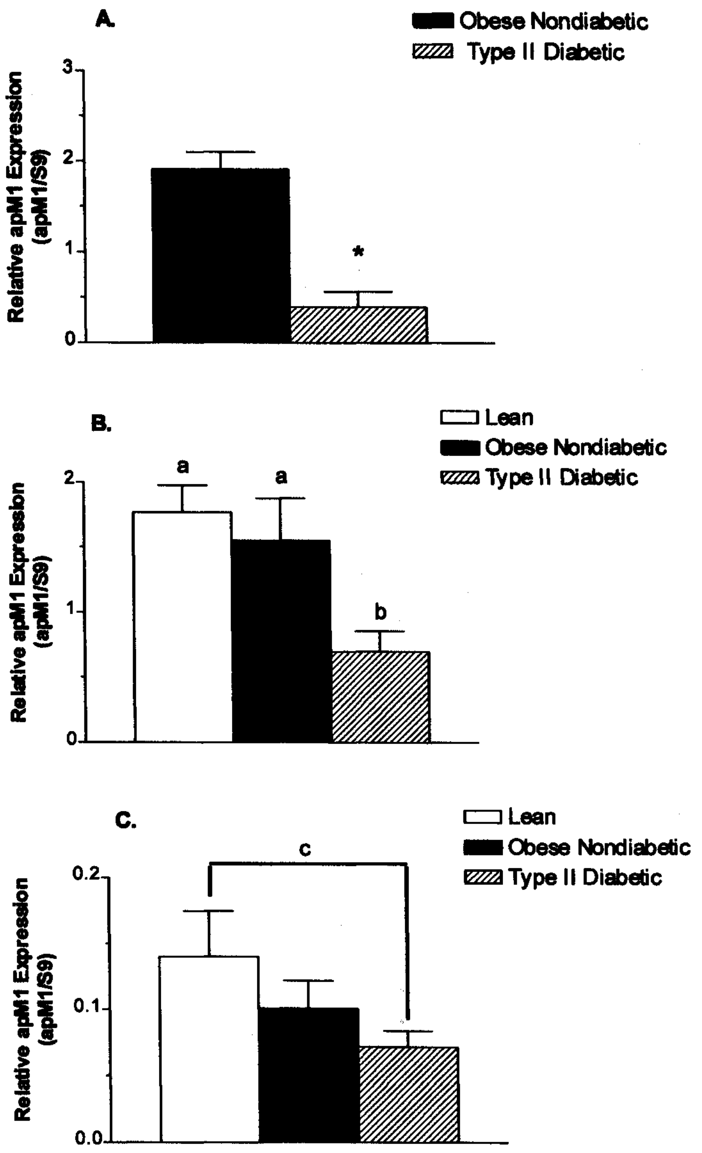

FIGURE 3 Semiquantitative RT-PCR analysis of apM1 expression in human adipose tissue. Expression of apM1 mRNA in omental adipose tissue isolated from the patients used in the generation and screening of the subtracted cDNA library (A). Expression of apM1 mRNA in omental adipose tissue isolated from lean, obese non-diabetic and obese Type 2 diabetic individuals (B). Expression of apM1 mRNA in subcutaneous adipose tissue isolated from lean, obese non-diabetic and obese Type 2 diabetic individuals (C). Analysis of amplified apM1 and S9 products was performed after 23 cycles and 27 cycles in omental and subcutaneous adipose tissue, respectively. These cycles were found to be within the exponential phase of the respective PCR reactions. * = $P < 0.05$ compared to obese non-diabetic individuals by Student's *t*-test. b = $P < 0.01$ compared to a groups by one-way ANOVA. c = not significant, $P < 0.1$ compared to lean individuals by one-way ANOVA. Data shown are shown as means ± S.E.M. for each group.

fold) from patients with Type 2 diabetes (Fig. 1). Sequencing #1738 and subsequent BLAST analysis revealed that this clone was identical to a recently cloned adipose-specific gene apM1.[10] Consistent with earlier reports on the murine orthologue Acrp30/AdipoQ,[8, 9] apM1 mRNA was readily detectable in mature human adipocytes but not in preadipocytes (Fig. 2). These data verify that apM1 is expressed in human adipocytes.

To confirm our findings from colony arrays, expression of apM1 was analyzed by semiquantitative RT-PCR. Consistent with our previous data, expression of apM1 was reduced 4.8 fold in the omental adipose tissue of Type 2 diabetic patients (normalized apM1 = 0.39 ± 0.17) compared to the non-diabetic obese subjects (normalized apM1 = 1.91 ± 0.19) used to generate the subtracted library (Fig. 3A). We next compared apM1 expression in omental adipose tissue from lean, obese non-diabetic, and obese Type 2 diabetic patients. Interestingly, expression of apM1 was reduced approximately 2.2–2.5 fold in omental adipose tissue from Type 2 diabetic patients (normalized apM1 = 0.70 ± 0.15, $n = 8$) compared to lean (normalized apM1 = 1.77 ± 0.21, $n = 6$) or obese non-diabetic (normalized apM1 = 1.55 ± 0.33, $n = 10$) individuals (Fig. 3B). In order to determine whether abnormalities in apM1 expression exist in other adipose depots, we analyzed apM1 in subcutaneous adipose tissue. As can be seen in Figure 3C, apM1 was reduced 2 fold in subcutaneous adipose tissue from diabetic patients (normalized apM1 = 0.07 ± 0.03, $n = 8$) compared to lean patients (normalized apM1 = 0.14 ± 0.03, $n = 7$), while obese non-diabetic individuals exhibited intermediate apM1 levels (normalized apM1 = 0.10 ± 0.02, $n = 9$). Thus, it appears that apM1 levels are reduced in both adipose depots in obese patients with Type 2 diabetes.

### DISCUSSION

In the present study we sought to identify genes that are differentially expressed in omental adipose tissue of patients with Type 2 diabetes. Interestingly, we identified apM1 from a small group of patients whose expression is reduced with diabetes. ApM1 (synonyms: Acrp30, AdipoQ, GBP28) encodes a 244 amino acid protein (known as adiponectin) that is most homologous to complement factor C1q. Moreover, the crystal structure [11] and gene organization [13] of apM1 indicate that this gene has many similarities to TNF$\alpha$. Previous studies have shown that the murine orthologue of apM1, Acrp30/AdipoQ, is expressed exclusively in adipose tissue. Consistent with these data, we found that apM1 mRNA is expressed only in the fully differentiated human adipocytes and not in preadipocytes. Therefore, it appears that apM1 encodes another cytokine secreted from adipocytes.

Unlike the other adipose-secreted cytokines (leptin, TNF$\alpha$, and PAI-1), Hu *et al.* (1996) reported that apM1/Acrp30/AdipoQ mRNA levels were reduced in subcutaneous adipose tissue of obese humans and in ob/ob mice. [9] Recently, a Japanese study reported that plasma levels of apM1 were negatively correlated with BMI. [12] Interestingly, the correlation between BMI and apM1 was stronger in male ($r = -0.71$) compared to female ($r = -0.51$) subjects. Moreover, apM1 levels were significantly higher in women compared to men, independent of adiposity. In this regard circulating apM1 is similar to leptin, which is also elevated in women compared to men per unit of fat mass. [14] These data suggest that gender-based differences exist in the regulation of apM1 gene expression.

In the patients used in the present study, we were unable to detect a significant change in apM1 expression in either omental or subcutaneous adipose tissue associated with obesity in the absence of frank diabetes. These findings contrast with an earlier study [9] that reported a reduction in apM1 mRNA in obese humans. Since the previous study employed a more accurate Northern blot analysis, one might speculate that the disparity in results was generated by our use of RT-PCR. However, we found that leptin mRNA levels were elevated 4–6 fold with obesity in our patients using RT-PCR under identical conditions (data not shown), validating our technique with data from previous studies. [15,16] A more likely source for the disparity in findings is the small number of patients used in this and previous [9]

studies. Moreover, our patients were women, which exhibit greater variability in apM1 levels in comparison with men.[12] Therefore, future studies should focus on expanding the numbers of patients analyzed, paying appropriate attention to the gender of the patients.

While apM1 secretion rate is increased by insulin[8] and apM1 mRNA is reduced in the insulin resistant ob/ob mouse,[9] we found no correlation in apM1 mRNA with fasting insulin ($r^2 = 0.15$). However, we found the most pronounced reduction in apM1 was in omental adipose tissue, the fat depot most strongly correlated with development of insulin resistance and Type 2 diabetes.[17–19] In addition, the chromosomal location of the apM1 gene is in a susceptibility locus for Familial Combined Hyperlipidaemia and polygenic Type 2 diabetes.[13] Based on these data, an intriguing consideration is that reduced apM1 levels somehow participate in the development of Type 2 diabetes. As the function and signaling properties of apM1 are presently unknown, future studies will be necessary to determine its role in the pathophysiology of obesity and Type 2 diabetes.

*Acknowledgments*

The authors wish to express their appreciation to Drs. G. L. Dohm and W. Pores at Eastern Carolina University Medical Center for organizing the patients, gathering the clinical data and collecting the adipose tissue samples.

### *References*

[1] Björntorp, P. (1997). Obesity and Diabetes Mellitus. In: *Diabetes Mellitus*, 5 edition, edited by Porte, D. and Sherwin, R. S., pp. 553–564, Appleton & Lange, Stamford, CT.

[2] Mohamed-Ali, V., Pinkney, J. H. and Coppack, S. W. (1998). Adipose tissue as an endocrine and paracrine organ, *Int. J. Obesity*, **22**, 1145–1158.

[3] Hotamisligil, G. S., Arner, P., Caro, J. F., Atkinson, R. L. and Spiegelman, B. M. (1995). Increased adipose tissue expression of tumor necrosis factor-alpha in human obesity and insulin resistance, *J. Clin. Invest.*, **95**, 2409–2415.

[4] Hotamisligil, G. S., Murray, D. L., Choy, L. N. and Spiegelman, B. M. (1994). Tumor necrosis factor alpha inhibits signaling from the insulin receptor, *Proc. Natl. Acad. Sci. USA*, **91**, 4854–4858.

[5] Schwartz, M. W., Baskin, D. G., Kaiyala, K. J. and Woods, S. C. (1999). Model for the regulation of energy balance and adiposity by the central nervous system, *Am. J. Clin. Nutr.*, **69**, 584–596.

[6] Shimomura, I., Funahashi, T., Takahashi, M., Maeda, K., Kotani, K., Nakamura, T., Yamashita, S., Miura, M., Fukuda, Y., Takemura, K., Tokunaga, K. and Matsuzawa, Y. (1996). Enhanced expression of PAI-1 in visceral fat: possible contributor to vascular disease in obesity, *Nat. Med.*, **2**, 800–803.

[7] Alessi, M. C., Peiretti, F., Morange, P., Henry, M., Nalbone, G. and Juhan-Vague, I. (1997). Production of plasminogen activator inhibitor 1 by human adipose tissue: possible link between visceral fat accumulation and vascular disease, *Diabetes*, **46**, 860–867.

[8] Scherer, P. E., Williams, S., Fogliano, M., Baldini, G. and Lodish, H. F. (1995). A novel serum protein similar to C1q, produced exclusively in adipocytes, *J. Biol. Chem.*, **270**, 26746–26749.

[9] Hu, E., Liang, P. and Spiegelman, B. M. (1996). AdipoQ is a novel adipose-specific gene dysregulated in obesity, *J. Biol. Chem.*, **271**, 10697–10703.

[10] Maeda, K., Okubo, K., Shimomura, I., Funahashi, T., Matsuzawa, Y. and Matsubara, K. (1996). cDNA cloning and expression of a novel adipose specific collagen-like factor, apM1 (AdiPose Most abundant Gene transcript 1), *Biochem. Biophys. Res. Comm.*, **221**, 286–289.

[11] Shapiro, L. and Scherer, P. E. (1998). The crystal structure of a complement-1q family protein suggests an evolutionary link to tumor necrosis factor, *Curr. Biol.*, **8**, 335–338.

[12] Arita, Y., Kihara, S., Ouchi, N., Takahashi, M., Maeda, K., Miyagawa, J., Hotta, K., Shimomura, I., Nakamura, T., Miyaoka, K., Kuriyama, H., Nishida, M., Yamashita, S., Okubo, K., Matsubara, K., Muraguchi, M., Ohmoto, Y., Funahashi, T. and Matsuzawa, Y. (1999). Paradoxical decrease of an adipose-specific protein, adiponectin, in obesity, *Biochem. Biophys. Res. Comm.*, **257**, 79–83.

[13] Schäffler, A., Orsó, E., Palitzsch, K. D., Büchler, C., Drobnik, W., Fürst, A., Schölmerich, J. and Schmitz, G. (1999). The human apM-1, an adipocyte-specific gene linked to the family of TNF's and to genes expressed in activated T cells, is mapped to chromosome 1q21.3-q23, a susceptibility locus identified for familial combined hyperlipidemia (FCH), *Biochem. Biophys. Res. Comm.*, **260**, 416–425.

[14] Hickey, M. S., Israel, R. G., Gartiner, S. N., Considine, R. V., McCammon, M. R., Tyndall, G. L., Houmard, J. A., Marks, R. H. and Caro, J. F. (1996). Gender differences in serum leptin levels in humans, *Biochem. Mol. Med.*, **59**, 1–6.

[15] Lonnqvist, F., Nordfors, L., Jansson, M., Thorne, A., Schalling, M. and Arner, P. (1997). Leptin secretion from adipose tissue in women. Relationship to plasma levels and gene expression, *J. Clin. Invest.*, **99**, 2398–2404.

[16] Lindgren, A. C., Marcus, C., Skwirut, C., Elimam, A., Hagenas, L., Schalling, M., Avert, M. and Lonnqvist, F. (1997). Increased leptin mRNA and serum leptin levels in children with Prader-Willi syndrome and nonsyndromal obesity, *Pediatr. Res.*, **42**, 593–596.

[17] Coon, P. J. Rogus, E. M., Drinkwater, D., Muller, D. C. and Goldberg, A. P. (1992). Role of body fat distribution in the decline in insulin sensitivity and glucose tolerance with age, *J. Clin. Endocrinol. Metab.*, **75**, 1125–1132.
[18] Carey, D. G., Jenkins, A. B., Campbell, L. V., Freund, J. and Chisholm, D. J. (1996). Abdominal fat and insulin resistance in normal and overweight women: Direct measurements reveal a strong relationship in subjects at both low and high risk of NIDDM, *Diabetes*, **45**, 633–638.
[19] Barzilai, N., She, L., Liu, B., Vuguin, P., Cohen, P., Wang, J. and Rossetti, L. (1999). Surgical removal of visceral fat reverses hepatic insulin resistance, *Diabetes*, **48**, 94–98.

